# Modification of Fibronectin by Non-Enzymatic Glycation Impairs K^+^ Channel Function in Rat Cerebral Artery Smooth Muscle Cells

**DOI:** 10.3389/fphys.2022.871968

**Published:** 2022-06-27

**Authors:** Yan Yang, Zahra Nourian, Min Li, Zhe Sun, Liping Zhang, Michael J. Davis, Gerald A. Meininger, Jianbo Wu, Andrew P. Braun, Michael A. Hill

**Affiliations:** ^1^ Dalton Cardiovascular Research Center, Columbia, MO, United States; ^2^ Department of Medical Pharmacology and Physiology, University of Missouri, Columbia, MO, United States; ^3^ Southwest Medical University, Luzhou, China; ^4^ Department of Physiology and Pharmacology, University of Calgary, Calgary, AB, Canada

**Keywords:** advanced glycation end-products (AGE), protein modification, K+ channels, extracellular matrix, fibronectin (FN), vascular dysfunction and damage, smooth muscle

## Abstract

Fibronectin (FN) enhances K^+^ channel activity by integrin-mediated mechanisms. As vascular smooth muscle (VSM) K^+^ channels mediate vasodilation, we hypothesized that modification of fibronectin, via advanced non-enzymatic glycation, would alter signaling of this extracellular matrix protein through these channels. Bovine FN (1 mg/ml) was glycated (gFN) for 5 days using methylglyoxal (50 mM), and albumin was similarly glycated as a non-matrix protein control. VSM cells were isolated from rat cerebral arteries for measurement of macroscopic K^+^ channel activity using whole cell patch clamp methodology. Pharmacological inhibitors, iberiotoxin (0.1 μM) and 4-aminopyridine (0.1 mM), were used to identify contributions of large-conductance, Ca^2+^-activated, K^+^ channels and voltage-gated K^+^ channels, respectively. Compared with baseline, native FN enhanced whole cell K^+^ current in a concentration-dependent manner, whereas gFN inhibited basal current. Furthermore, native albumin did not enhance basal K^+^ current, but the glycated form (gAlb) caused inhibition. gFN was shown to impair both the Kv and BK_Ca_ components of total macroscopic K^+^ current. Anti-integrin α5 and β1 antibodies attenuated the effects of both FN and gFN on macroscopic K^+^ current at +70 mV. Consistent with an action on BK_Ca_ activity, FN increased, whereas gFN decreased the frequency of spontaneous transient outward current (STOCs). In contrast, gAlb inhibited whole cell K^+^ current predominantly through Kv, showing little effect on STOCs. A function-blocking, anti-RAGE antibody partially reversed the inhibitory effects of gFN, suggesting involvement of this receptor. Further, gFN caused production of reactive oxygen species (ROS) by isolated VSMCs as revealed by the fluorescent indicator, DHE. Evoked ROS production was attenuated by the RAGE blocking antibody. Collectively, these studies identify ion channel-related mechanisms (integrin and ROS-mediated) by which protein glycation may modify VSMC function.

## 1 Introduction

Vascular dysfunction remains a major cause of the increased morbidity and mortality associated with diabetes mellitus. For example, diabetes is associated with increased rates of coronary artery disease, heart failure, stroke and microvascular dysfunction (Grundy et al., 1999; Einarson et al., 2018). Amongst the biochemical changes occurring in this common metabolic disorder, matrix protein glycation, as well as matrix protein deposition, potentially affects all cellular elements of the vascular wall. As many molecules residing in the extracellular matrix (ECM) are now accepted to participate in active signaling pathways in vascular cells (Davis et al., 2001; Davis et al., 2002; Martinez-Lemus et al., 2003; Malinin et al., 2012; Finney et al., 2017; Liu and Lin, 2021) it is likely that matrix protein modification impacts normal vessel function.

ECM proteins undergo non-enzymatic glycation (initially through Schiff base and Maillard reactions) with the eventual formation of advanced glycation end-products (AGEs) (Monnier et al., 2005). For example, glycation of fibrillar and type IV collagens, laminin, fibronectin and vitronectin has been reported (Tarsio et al., 1987; Monnier et al., 2005); (McDonald et al., 2009). Typically, this modification of proteins occurs through the initial interaction of a reducing sugar, such as glucose and glucose-6-phosphate, with free amino groups of proteins (although similar processes occur in other macromolecules). Commonly this reaction occurs on epsilon amino groups of lysine and the free amino group of arginine residues as well as on the amino terminus of proteins.

Modification of proteins through glycation and cross-linking reactions has the potential to alter the secondary and tertiary structural characteristics of proteins (Povey et al., 2008). In doing so, it is conceivable that these alterations impact protein-protein interactions that are dependent, in part, on protein conformation. In regard to matrix proteins, this may affect how these proteins interact with cellular matrix binding proteins (for example via cell surface integrins) to affect cell signaling and adhesive events (Wu et al., 2001; Yang et al., 2009; Hong et al., 2012; Sun et al., 2012; Zhu et al., 2012). In this context, we have reported that glycation of fibronectin alters its cell binding/adhesion properties as assessed by atomic force microscopy (AFM) (Dhar et al., 2017). Further, as matrix proteins exert numerous other cellular functions, including regulation of cell growth and migration, structural interactions, growth factor binding and as determinants of the physical properties of tissues, these may be impacted by glycation-induced changes in conformation.

Potassium channels play an important role in the setting of smooth muscle cell membrane potential and vascular tone. Through these properties, K^+^ channels further regulate voltage-gated Ca^2+^ channels and the contractile state of resistance arteries (Nelson and Quayle, 1995; Ledoux et al., 2006; Hill et al., 2010; Tykocki et al., 2017). At the cellular level it has been shown that K^+^ channels can be modulated by fibronectin binding to cell surface integrins (Wu et al., 2008; Gui et al., 2010; Yang et al., 2010). Specifically, it has been demonstrated that fibronectin increases the activity of the large conductance, Ca^2+^ activated, K^+^ channel (BK_Ca_) *via* binding to α_5_β_1_ integrins (Wu et al., 2008; Gui et al., 2010; Yang et al., 2010). In addition, physical forces transmitted through fibronectin have been proposed to link skeletal muscle activity to functional hyperemia, although in this case, a non-integrin-mediated binding was additionally implicated (Hocking et al., 2008). The present studies therefore aimed to determine whether advanced glycation of fibronectin impacts K^+^ channel activity in isolated arterial vascular smooth muscle cells. Cells were obtained from the cerebral arteries due to the potential relevance to cerebrovascular dysfunction in diabetes and to extend our previous studies on K^+^ channels in this tissue (Yang et al., 2009; Yang et al., 2013). As the formation of AGEs may also activate signaling via the receptor for AGEs (RAGE) (Yan et al., 2003; Xu et al., 2010; Rai et al., 2012; Manigrasso et al., 2014), the present studies also examined the role of this pathway in altered K^+^ channel activity. Macroscopic VSMC K^+^ channel current was examined using the whole cell patch clamp technique together with pharmacological and inhibitory antibody approaches for characterizing underlying signaling mechanisms. As any effects of glycated fibronectin could conceivably occur through both cell surface integrins and RAGE, we also examined the functional effects of glycated albumin as a non-integrin binding protein. Additional studies using fluorescence confocal microscopy were performed to measure ROS production as an indicator of acute AGE signaling acting via RAGE.

## 2 Material and Methods

The studies used male Sprague–Dawley rats weighing 180–280 g. Prior to experiments, animals were housed in a temperature, humidity and light controlled animal facility with free access to standard rat chow and drinking water. All procedures were approved by the Animal Care and Use Committee of the University of Missouri, Missouri, United States. Rats were anaesthetized by intraperitoneal injection of sodium pentobarbital (Nembutal, 100 mg/kg). Following euthanasia by anesthetic overdose, a craniotomy was performed, and the brain removed and placed in a cooled dissection chamber. Segments of anterior cerebral artery and posterior communicating artery, from the Circle of Willis, were then micro-dissected.

### 2.1 Isolation of Vascular Smooth Muscle Cells From Small Cerebral Arteries

Smooth muscle cells were enzymatically isolated as previously described (Yang et al., 2010; Zhu et al., 2012). Briefly, dissected vessel segments were transferred to a 1 ml tube of low-Ca^2+^ physiological salt solution (PSS) containing (in mm): NaCl, 144; KCl, 5.6; CaCl_2_, 0.1; MgCl_2_, 1.0; Na_2_HPO_4_, 0.42; Hepes, 10; sodium pyruvate, 2; and 1 mg ml^−1^ BSA at room temperature (RT) for 10 min. Vessels were then exposed to a two-step digestion process: 1) 15 min incubation in PSS (37°C) containing 0.6 mg ml^−1^ papain and 1.8 mg ml^−1^ DTT; and 2) 5–6 min incubation in PSS with 0.7 mg ml^−1^ type F collagenase and 0.4 mg ml^−1^ type H collagenase. Following enzyme treatment, vessel pieces were washed repeatedly with fresh ice-cold low-Ca^2+^ PSS and triturated with a fire-polished pipette. Liberated smooth muscle cells were stored in ice-cold PSS for use within 4 h.

### 2.2 Whole Cell Patch Clamping for Measurement of K^+^ Currents

An EPC-10 USB amplifier (HEKA, Germany) was controlled via a Dell computer using Patchmaster software. Igor Pro (Wavemetrics, Inc, Lake Oswego, OR, USA) was used for data analysis. The standard whole-cell patch clamp configuration (Hamill et al., 1981) was used to record the whole cell outward K^+^ currents. The currents were activated by voltage step pulses (from -70 to +70 mV, in 20 mV increments, duration of 300 ms) delivered from a holding potential of −70 mV as previously described (Yang et al., 2010; Zhu et al., 2012; Yang et al., 2013). In some experiments, the currents were activated by a voltage ramp (from −70 to +80 mV). Micropipettes were pulled from borosilicate glass tubing (Corning 8161; ID, 1.2 mm; OD, 1.5 mm; Warner Instruments Corp, Hamden, CT, United states) using a Sutter P-97 electrode puller (Sutter Instrument Co., Novato, CA, United States). Pipette tip resistances ranged from 3–5 MΩ when filled with standard intracellular solution. The series resistance (*<*10 MΩ) was compensated to minimize the duration of the capacitive surge. Subtraction of leak currents was not performed. The whole cell currents were sampled at 2–5 kHz and filtered at 2.9 kHz *via* a four-pole low-pass Bessel filter. Currents were normalized to cell capacitance and are expressed as picoampere per picofarad (pA/pF). The average cell capacitance of cerebral SMCs was 16.7 ± 2.2 pF.

For whole-cell recordings, the bath solution contained (in mM) 140 NaCl, 5.4 KCl, 1.5 CaCl_2_, 1 MgCl_2_, 10 glucose, 10 HEPES, 2 sodium pyruvate (pH 7.4). The pipette solution contained (in mM) 140 KCl, 5 NaCl, 2 EGTA, 3 Mg-ATP, 10 HEPES (pH7.2); CaCl_2_ was added to bring free [Ca^2+^] to a computed 400 nM. Mg-ATP was included to inhibit ATP-sensitive K^+^ channels and provide substrate for energy-dependent processes. Where addition of other reagents to the bath and/or pipette solutions was required, details are given in specific protocols and figure legends. All experiments were performed at room temperature. The relative contributions of the large conductance, Ca^2+^-activated, K^+^ channel (BK_Ca_) and voltage-gated K^+^ channels (K_V_) were determined using pharmacological inhibitors. BK_Ca_ current was identified using iberiotoxin (IBTX; 10^−7^ M) and K_V_ current by 4-aminopyridine (4-AP; 10^−4^ M).

Spontaneous transient outward currents (STOCs) were recorded as an index of BK_Ca_ channel activation. STOCs typically represent clusters of BK_Ca_ channel openings driven by local release of Ca^2+^ sparks from the adjacent sarcoplasmic reticulum (Zhuge et al., 2000; Yang et al., 2009). STOCs were recorded at a holding potential of +30 mV. For analysis of STOC frequency, a 5 pA threshold amplitude was applied for detection.

### 2.3 Preparation and Characterization of Glycated Fibronectin and Albumin

Glycation of human plasma fibronectin (gFN) and bovine serum albumin (gALB) was performed by incubation with 50 mM methylglyoxal for 5 and 7 days respectively at 37°C under sterile conditions (Naser et al., 2013; Dhar et al., 2017). Methylglyoxal was used as a physiologically relevant glycating agent (Cantero et al., 2007) while promoting glycation at a faster rate than does glucose. Dialysis was performed against sodium phosphate buffer for 24 h to remove excess glycating agent. Control proteins underwent similar treatment, but without the glycating agent. Endotoxin levels were determined to be < 0.02 ng/ml using a limulus amoebocyte assay (Lonza, Walkersville, MD). The final protein content was analyzed using BCA protein assay kit (Thermofisher Scientific, Waltham, MA). AGE was evident by the brown color of gALB and gFN solutions and the production of AGEs formed *via* the Maillard reaction was confirmed by an increase in intrinsic fluorescence and absorbance (Naser et al., 2013; Dhar et al., 2017). Using a micro-plate reader (Synergy HT, Bio-Tek Inc.) fluorescence was measured at an excitation wavelength of 360 nm and an emission wavelength of 460 nm. The increase in intrinsic absorbance was measured at 280 nm (Naser et al., 2013; Dhar et al., 2017). For AGE-BSA and AGE-FN, the fluorescence and absorbance were measured at a final protein concentration of 1 mg/ml and 0.65 mg/ml respectively.

### 2.4 Demonstration of RAGE Expression

#### 2.4.1 RNA Isolation and End-Point PCR Protocol

To demonstrate RAGE mRNA expression, total RNA was prepared from rat cerebral arteries and enzymatically isolated smooth muscle cells using a Melt Total Nucleic Acid isolation kit (Life Technology, CA) and a RNeasy Plus Mini Kit (Qiagen, CA), respectively, following the manufacturer’s instructions. Rat lung total RNA was also purified as a positive control for RAGE mRNA expression (Srikanth et al., 2011). To minimize contamination with genomic DNA, all samples were subjected to DNase digestion. The concentration and purity of RNA for each sample was determined by UV absorbance using a Nanodrop ND-1000 spectrophotometer (ThermoFisher Scientific, Wilmington, DE). Equal amounts of total RNA extracts were reverse-transcribed into a single strand cDNA using the Superscript III First-Strand synthesis system (Invitrogen, CA) according to the manufacturer’s instructions.

End-point PCR was performed using the Go Taq Flexi DNA Polymerase method (Promega, Madison WI). Due to the possibility of splice variant expression (Srikanth et al., 2011) two sets of primers (A and B) were designed as follows to amplify RAGE mRNA:

Primer set (A): forward5′-GTGAATCCTGCCTCTGAACTC-3′ and reverse5′-ACTGTCCCTTTGCCATCAG-3’ (amplicon = 137 bp).

Primer set (B) forward5′-GGTACTGGTTCTTGCTCTGTG-3′ and reverse5′-ATTCTAGCTTCTGGGTTGGC-3’ (amplicon = 122 bp).

End-point PCR protocols were performed as follows: pre-heating at 95°C for 2 min, 35 cycles of three-step cycling of denaturation at 95°C for 20 s, annealing at 56°C for 20 s, and extension for 40 s at 72°C followed by final extension of 5 min at 72°C. As shown in Supplementary Figure S1, electrophoretic separation (2% agarose) of lung samples showed PCR products of sizes 137 bp (primer set A) and 122 bp (primer set B). Sequencing results confirmed the transcripts to be RAGE mRNA (accession# NM_053336.2). For cerebral arteries and VSMCs, two PCR products were detected using primer set (A) which were confirmed as RAGE variants by sequencing. A larger PCR product, 320 bp, was detected for both cerebral arteries and VSMCs using primer set (B). Sequencing confirmed this product to be a previously described RAGE mRNA variant (accession # GU164715.1). Collectively these data demonstrate the existence of mRNA for RAGE in small cerebral arteries and further suggest the presence of known splice variants (note that the functional significance of these variants was not considered in the present studies).

### 2.5 Measurement of ROS Production *Via* Dihydroethidium Fluorescence and Confocal Microscopy

Single VSMC ROS production was measured following treatment with either FN or gFN using dihydroethidium (DHE; 5 μM) fluorescence confocal microscopy. Changes in fluorescence were monitored over a 30 min time period, at room temperature, using a Leica confocal microscope. Images were collected using a ×63 water-immersion, 1.2 numerical aperture, objective lens and the following parameters: excitation wavelength of 514 nm, emission detection wavelength of 600–700 nm (Staiculescu et al., 2014). As positive controls, ROS production was also examined in response to angiotensin II [Ang II, 10^−7^ M (Touyz and Schiffrin, 2001)] and antimycin (10^−5^ M) (Gao et al., 2009). Un-stimulated cells treated with DHE alone and DMSO were used as negative/time controls.

### 2.6 Chemicals and Reagents

Unless stated otherwise, all chemicals and reagents were purchased from the SigmaAldrich Chemical Company (St Louis, MO).

### 2.7 Statistical Methods

Statistical analysis was performed using Excel and Prism software. Results are expressed as mean ± SEM. Each data set is expressed as n = number of cells obtained from 4-6 individual cell preparations; one animal used per preparation. Comparisons between two groups were made using the Student’s t-test. Multiple comparisons were performed using one or two-way analysis of variance (ANOVA) followed by a Tukey *post hoc* test. Statistical significance was assumed at *p* < 0.05.

## 3 Results

### 3.1 Acute Effects of Native and Glycated Fibronectin on K^+^ Channel Currents

**FIGURE 1:**
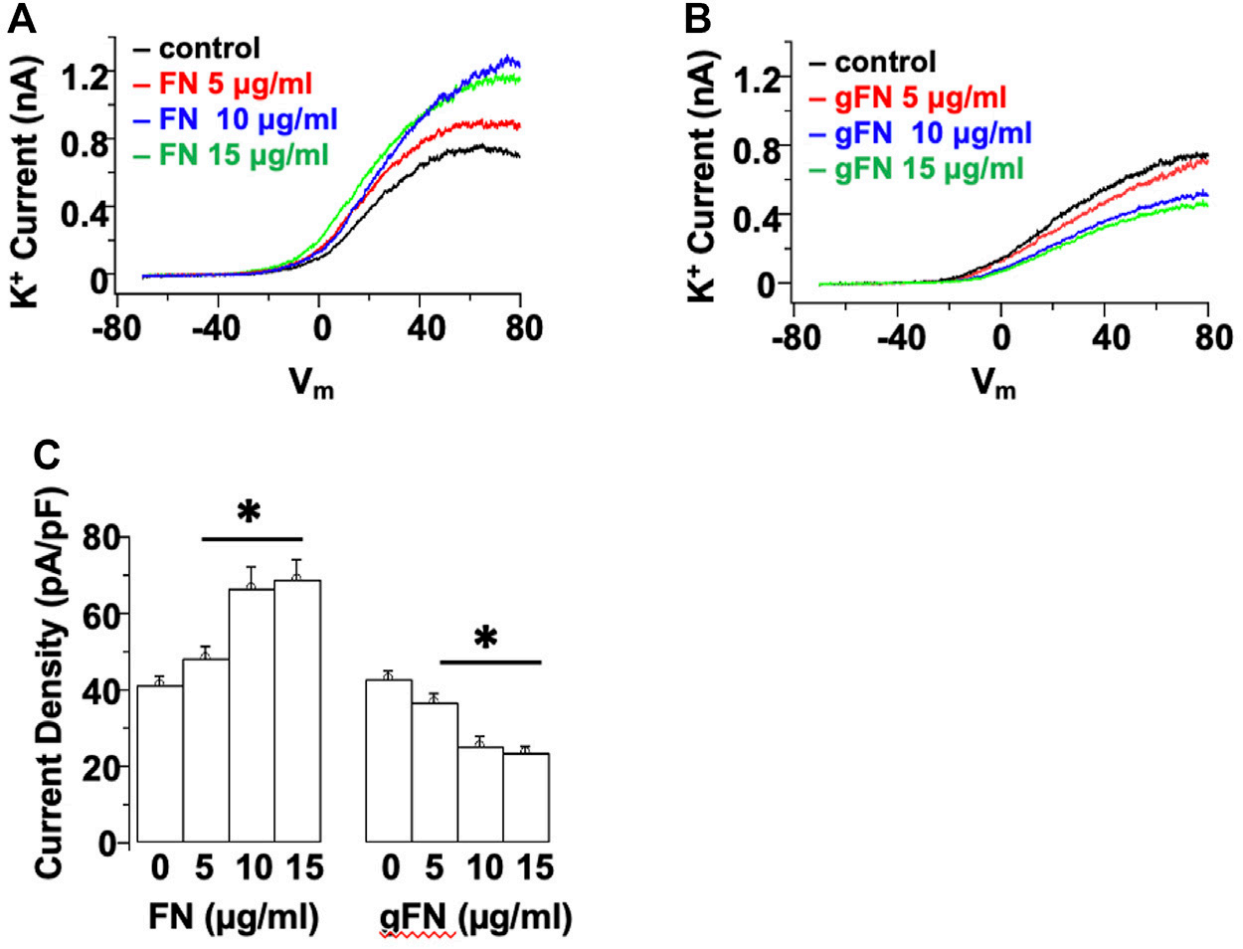
Acute application of native FN evokes a concentration—dependent increase in whole cell K^+^ current, whereas gFN causes a concentration—dependent inhibition of K^+^ currents. Panels **(A,B)** show representative I–V responses in single cerebral VSMCs in the presence of increasing concentrations of native and modified FN. Whole cell currents were evoked by a voltage ramp from −70 to +80 mV and are displayed as the averages of three consecutive ramps under each condition. **(C)** shows group data at +70 mV (*n* = 12; mean ± SEM). *p < 0.05 compared to control response in the absence of FN or gFN.

Example tracings showing the effect of control fibronectin (FN) and glycated FN (gFN) on K^+^ currents are displayed in Figures 1A,B. Consistent with previous studies in freshly isolated rat cremaster VSMCs (Wu et al., 2008) and HEK 293 cell expression systems (Yang et al., 2010), fibronectin evoked a concentration—dependent (5—15 μg/ml) increase in cerebral VSM whole cell K^+^ current (Figures 1A,C). In contrast, glycated fibronectin caused a concentration—dependent (5—15 μg/ml) inhibition of K^+^ current (Figures 1 B,C). For example, at a ramp potential of +70 mV, 15 μg/ml gFN caused a 53 ± 2% reduction in total K^+^ current while a similar concentration of FN caused a 183 ± 3% increase in K^+^ current (Figure 1C). Acute application of the glycated proteins did not cause any observable change in cellular morphology (Supplementary Figure S2).

**FIGURE 2:**
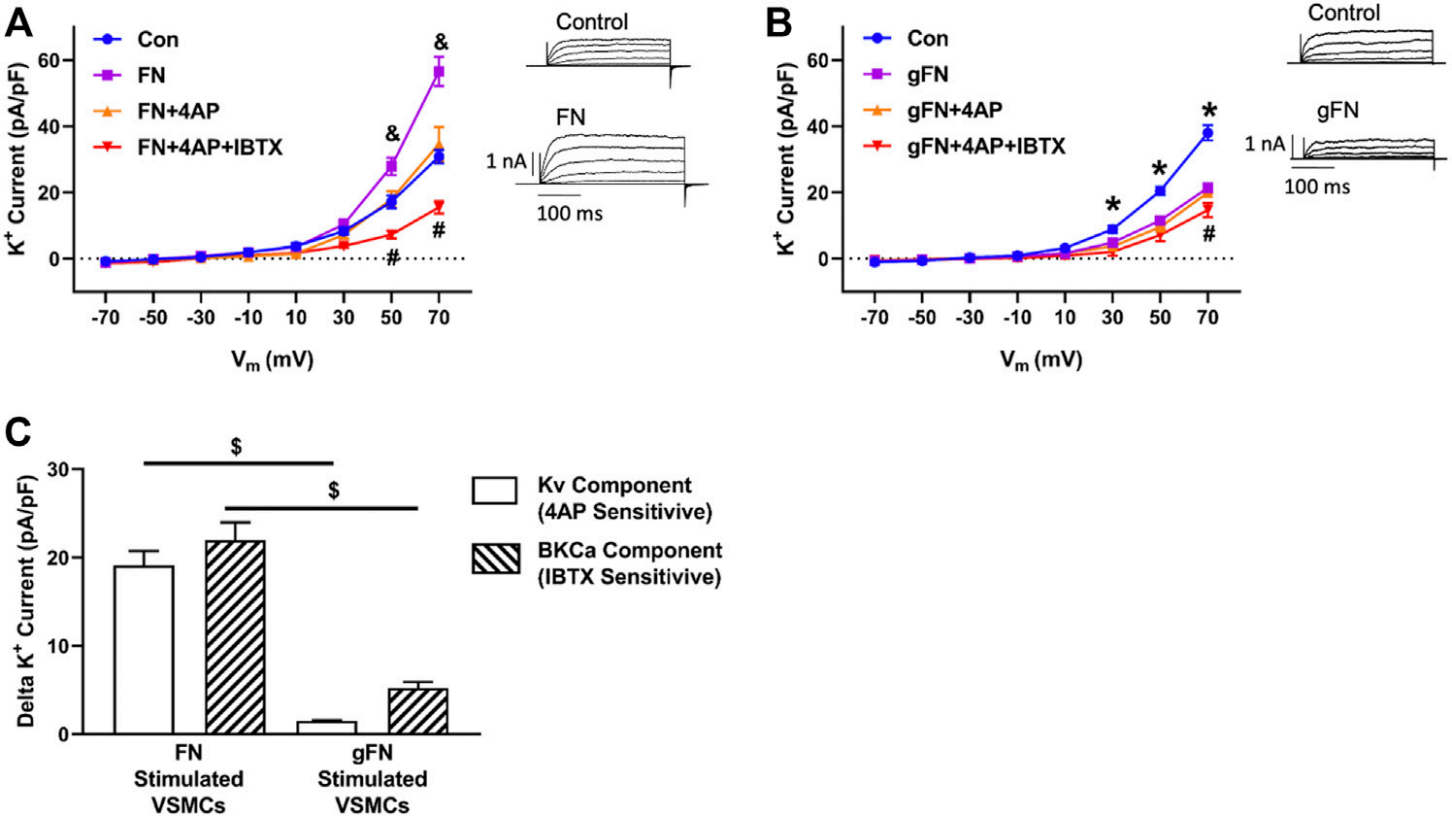
Effects of FN and gFN on major components of whole cell K^+^ current. Panel **(A,B)** Effect of FN (*n* = 12–15) and gFN (15 μg/ml; *n* = 26), respectively on whole cell K^+^ currents under control conditions and in the presence of selective K^+^ channel inhibitors. Components of the total K^+^ current were isolated by 4-AP (0.1 mM) to inhibit Kv channels and IBTX (0.1 μM) to block BK_Ca_ activity. The inserts display representative whole cell K+ currents recorded under control conditions and in the presence of either FN or gFN. & *p* < 0.05, FN vs. other conditions *p < 0.05, Control vs. gFN ± channel inhibitors; #*p* < 0.05, FN or gFN + both channel inhibitors vs. other conditions. Panel **(C)** illustrates that gFN inhibits the K_V_ and BK_Ca_ channel activities to differing degrees whereas the potentiating effect that fibronectin exerts on whole cell K^+^ current involves similar contributions from both Kv and BK_Ca_ channels. Total IBTX and 4-AP sensitive currents were obtained from panels **(A,B)** by calculating the difference between current amplitudes recorded at +70 mV in the presence of either FN or gFN alone vs currents recorded following addition of either 4-AP or IBTX. $ *p* < 0.05, FN vs. gFN. Bar graphs are presented as mean ± SEM for data at +70 mV.

To determine the involvement of select classes of K^+^ channels in the response to FN, additional recordings were performed in the presence of the BK_Ca_ channel inhibitor, IBTX (10^−7^ M), and the broad-spectrum inhibitor of K_V_ channels, 4-AP (10^−4^ M) (Figure 2). At a potential of +70 mV and in the presence of FN, Kv channel activity (i.e., 4-AP-sensitive component) contributed 19.7 ± 1.8 pA/pF to the whole cell outward K^+^ current, whereas BK_Ca_ channel activity (i.e., IBTX-sensitive component) contributed 21.9 ± 2.1 pA/pF to the total whole cell K^+^ current (Figure 2C). In contrast, in the presence of gFN, addition of 4-AP decreased the magnitude of the remaining whole cell K^+^ current by only 1.5 ± 0.6 pA/pF (i.e., contribution of remaining K_v_ channel activity), whereas IBTX evoked a further decrease of 5.2 ± 0.7 pA/pF (i.e., contribution of remaining BK_Ca_ channel activity (Figure 2C).

**FIGURE 3:**
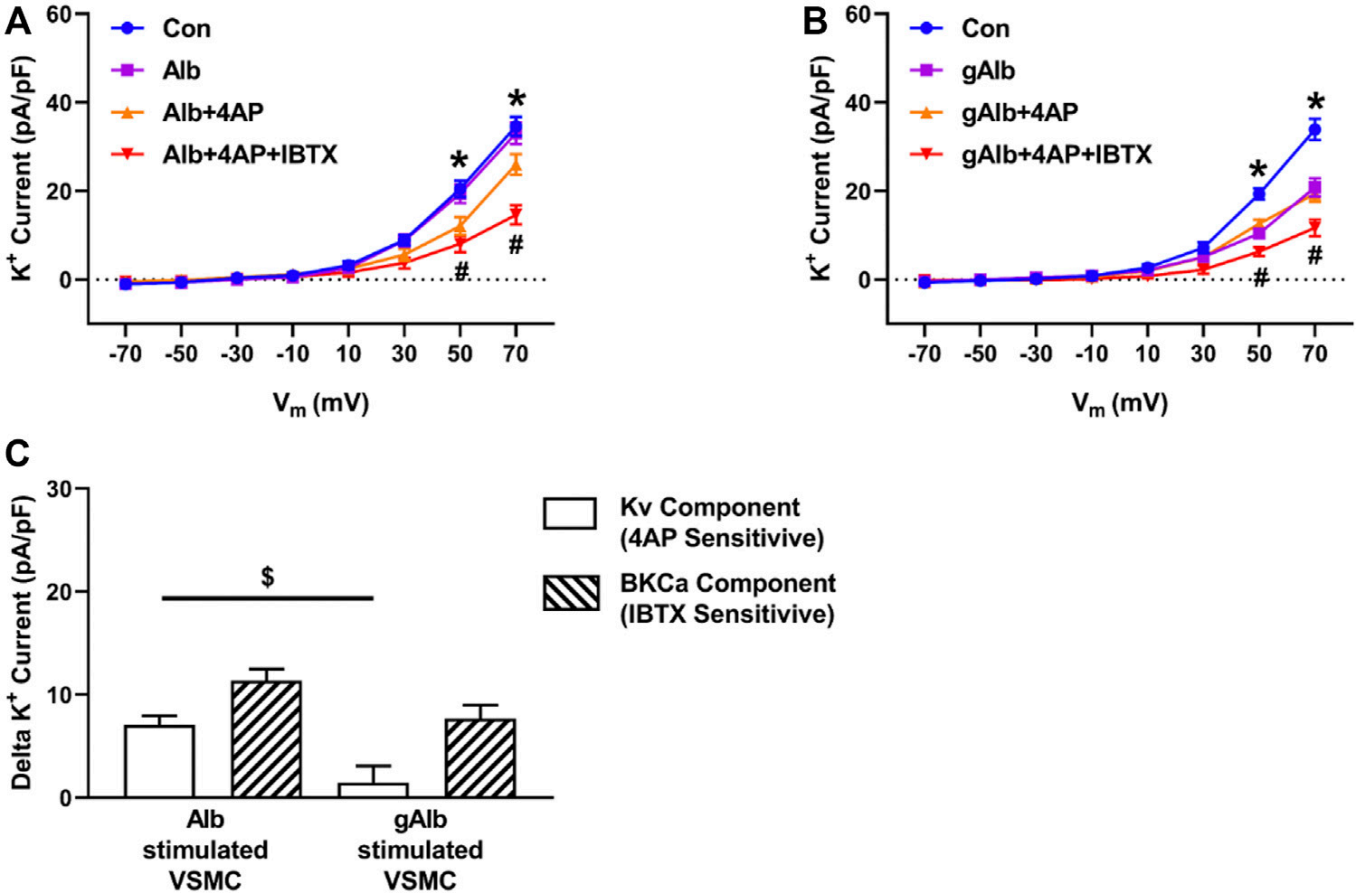
Effects of Alb and gAlb on major components of whole cell K^+^ current. Panel **(A,B)** Effect of Alb (*n* = 8) and gAlb (15 μg/ml; *n* = 7), respectively on whole cell K^+^ currents under control conditions and in the presence of selective K^+^ channel inhibitors. Components of the total K^+^ current were isolated by 4-AP (0.1 mM) to inhibit Kv and IBTX (0.1 μM) to block BK_Ca_. *p < 0.05, Control vs. Alb+4AP or gAlb+4AP; # all groups vs. Alb+4AP + IBTX or gAlb+4AP + IBTX. Panel **(C)** illustrates that gAlb inhibits both the K_V_ and BK_Ca_ components of whole cell K^+^ current, calculated as 4-AP and IBTX sensitive currents from panels **(A,B)** by subtracting current amplitudes in the presence of either Alb or gAlb currents from those observed following bath addition of 4-AP (0.1 mM) and IBTX (0.1 μM). Bar graphs are presented as mean ± SEM for data at +70 mV *p < 0.05.

To examine whether protein glycation, *per se,* impairs K^+^ channel function, the effect of gALB on whole cell K^+^ current was determined. Unlike fibronectin, native albumin did not significantly increase K^+^ current from baseline (Figure 3A). In contrast, gALB (15 μg/ml) caused a significant decrease in whole cell K^+^ current by 29.4% ± 1.8% compared with control/native albumin (Figure 3B). In terms of component currents, gALB caused a decrease in both the K_V_ and BK_Ca_ channel activities with the effect on Kv being particularly evident. (Figure 3C).

### 3.2 Acute Effects of Native Protein, Glycated Fibronectin and Glycated Albumin on STOC Frequency

**FIGURE 4:**
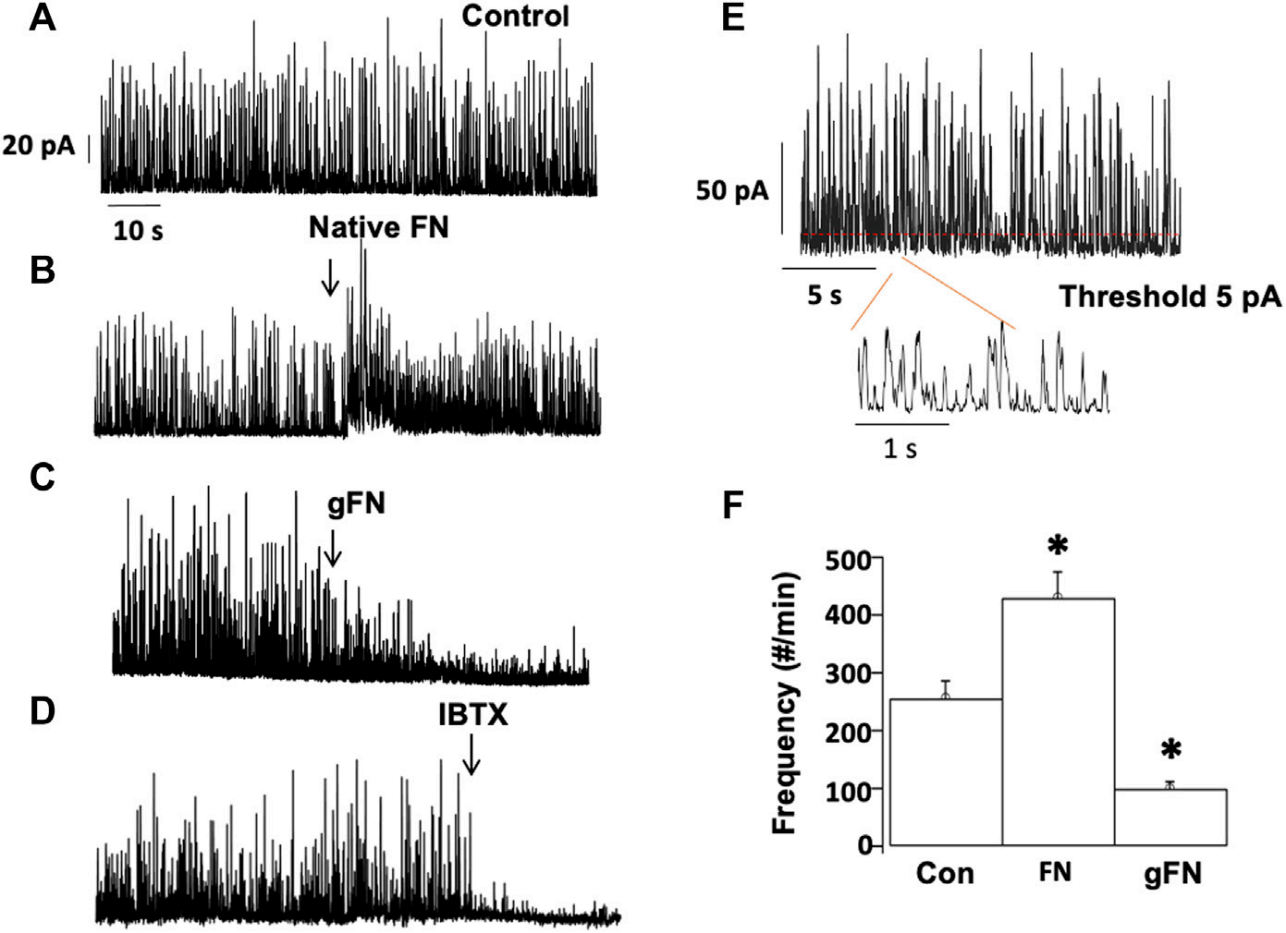
gFN decreases the frequency of STOCs in isolated cerebral vascular SMCs. Outward currents **(A)** were stimulated by native fibronectin **(B)**, while being attenuated by glycated fibronectin (gFN) **(C)**. STOC activity, dependent on BK_Ca_ channel opening, was inhibited by iberiotoxin (IBTX, 0.1 μM; **(D)**. Panel **(E)** illustrates the analytical method for quantification of STOC frequency and group data are shown in panel **(F)**. Recordings were made at a holding potential of +30 mV, *n* = 10–16 cells. The bar graph is presented as mean ± SEM; *p < 0.05 vs control.

As a further index of BK_Ca_ activity, spontaneous transient outward currents (STOCs) were recorded in the absence and presence of the various protein preparations. STOCs reflect the activity of clusters of BK_Ca_ channel openings. Consistent with a stimulatory effect of the native protein on whole cell BK_Ca_ channel activity (see Figure 2C), fibronectin (15 μg/ml) caused an acute and significant increase in STOC activity (compare example tracings in Figures 4A,B). In contrast, gFN (15 μg/ml) caused a significant decrease in STOC activity (compare example tracings in Figures 4A,C). Consistent with reports in the literature, STOC activity was abolished following addition of the specific BK channel inhibitor iberiotoxin (IBTX) (Figure 4D). STOC frequencies were quantified by setting a threshold current of 5 pA to detect individual STOCs (Figure 4E) and group data are shown in Figure 4F. FN increased STOC frequency to approximately 170% of control while gFN suppressed frequency to approximately 40% (Figure 4F). Caffeine (100 μM) applied to cells either before or after exposure to gFN evoked robust STOC activity, demonstrating that the SR Ca^2+^ stores in voltage-clamped cells remained intact and were not acutely altered by gFN (Supplementary Figure S3).

In contrast to FN, native ALB (15 μg/ml) did not cause a detectable alteration in STOC frequency (Supplementary Figure S4). gALB (15 μg/ml) produced a relatively modest inhibition of STOC frequency (to 80% of the control frequency;Supplementary Figure S4), consistent with its smaller effect on whole cell K^+^ current density as compared with gFN (see Figures 2, 3).

### 3.3 Integrin Involvement in Fibronectin—Mediated Enhancement of K^+^ Channel Current

**FIGURE 5:**
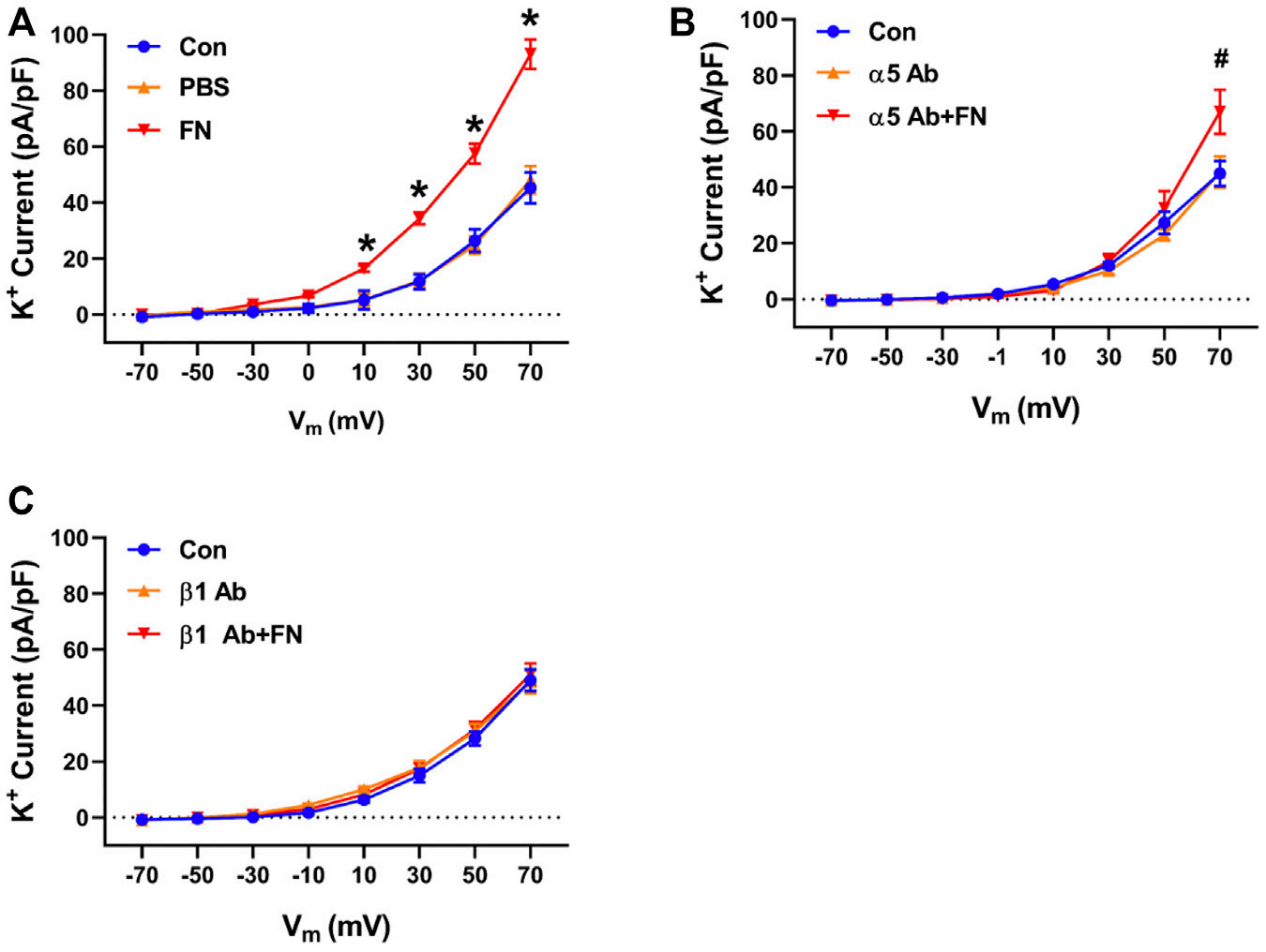
A function blocking antibody (Ab) versus the integrin subunit α5 or β1 interferes with the stimulatory effect of native FN on K^+^ currents of cerebral VSMCs. Panel **(A)**, current—voltage relationship under control/buffer (PBS) conditions. Panel **(B)**, current—voltage relationships in the presence of an α5 integrin antibody alone and α5 antibody plus FN. Panel **(C)**, current—voltage relations in the presence of a β1 integrin antibody alone and β1 antibody plus FN; *n* = 12–16 cells in each group. Results are shown as mean ± SEM. *p < 0.05 control and PBS vs. FN; #*p* < 0.05 control and α5 Ab vs. α5 Ab + FN.

**FIGURE 6:**
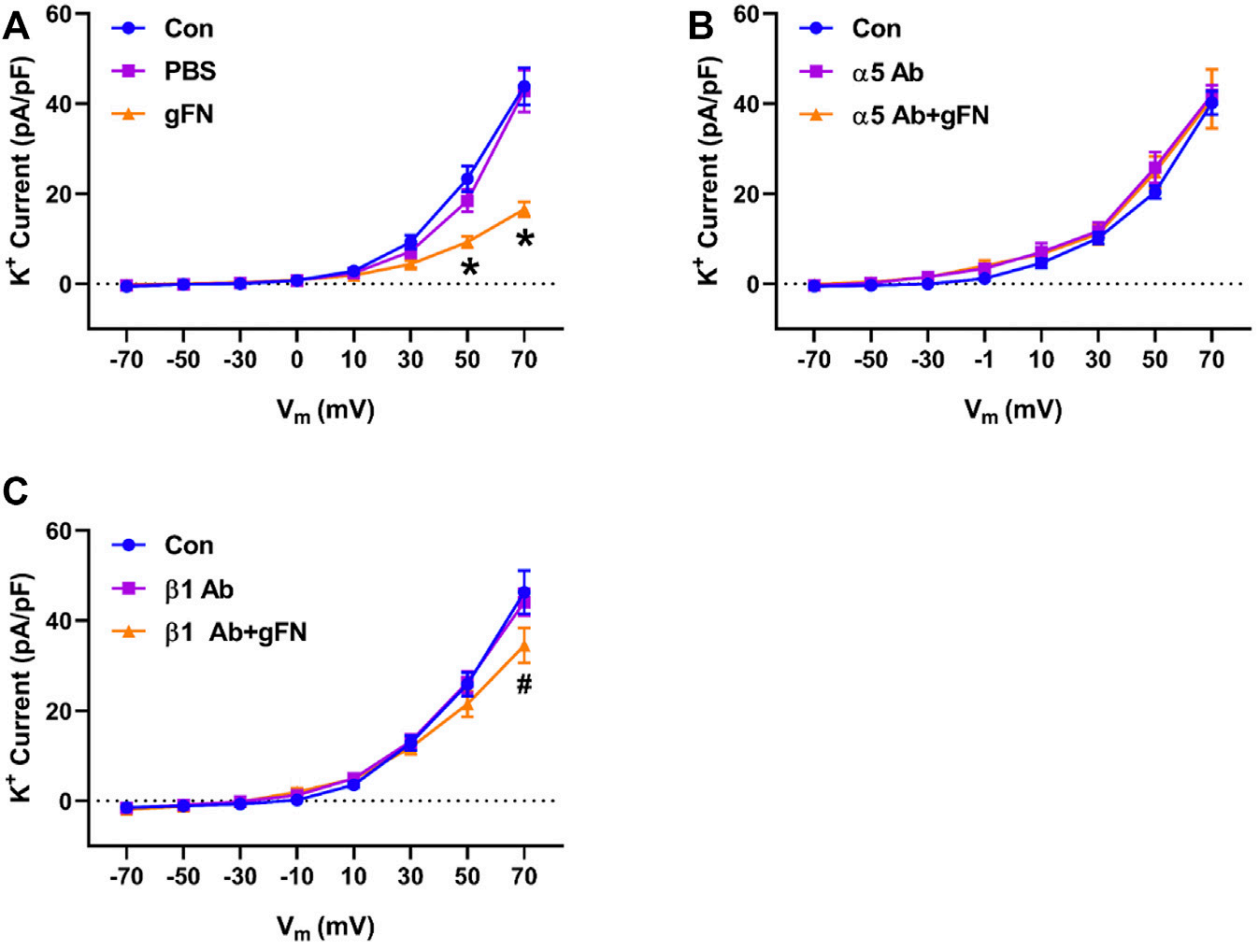
A function-blocking antibody versus either integrin α5 or β1 subunits blocks the gFN effect on whole cell K^+^ currents of cerebral VSMCs. Panel **(A)**, current—voltage relationship under control/buffer (PBS) conditions. Panel **(B)**, current—voltage relationships in the presence of α5 antibody (Ab) alone and α5 antibody plus gFN. Panel **(C)**, current-voltage relations in the presence of β1 antibody alone and β1 antibody plus gFN; *n* = 12 cells in each group. Results are shown as mean ± SEM. *p < 0.05 control and PBS vs. gFN; #*p* < 0.05 control and β1 Ab vs. β1 Ab + gFN.

Previous studies have reported that fibronectin-stimulated integrin signaling can modulate ion channel activity in vascular smooth muscle cells (Wu et al., 2008; Gui et al., 2010; Yang et al., 2010). These findings prompted us to examine the effects of function blocking α5 and β1 anti-integrin antibodies on the modulation of K^+^ currents by FN and gFN (Figures 5,6). Treatment of cells with either the α5 or β1 anti-integrin antibodies caused attenuation of the current enhancing effects of native fibronectin (Figure 5). Similarly, these same function-blocking antibodies to α5 and β1 integrins attenuated the inhibitory effects of gFN on whole cell K^+^ currents (Figure 6).

### 3.4 Effect of Function Blocking Anti RAGE Antibody on Glycated Fibronectin—Mediated Impairment of K^+^ Channel Currents

The expression of RAGE in small cerebral arteries and freshly isolated cerebral artery smooth muscle cells was first verified by isolation of mRNA by endpoint PCR and sequencing of the PCR product. Using two distinct primer sets, mRNA for previously described RAGE variants (accession numbers NM_053336.2 and GU164715.1) were identified (Supplementary Figure S1).

**FIGURE 7:**
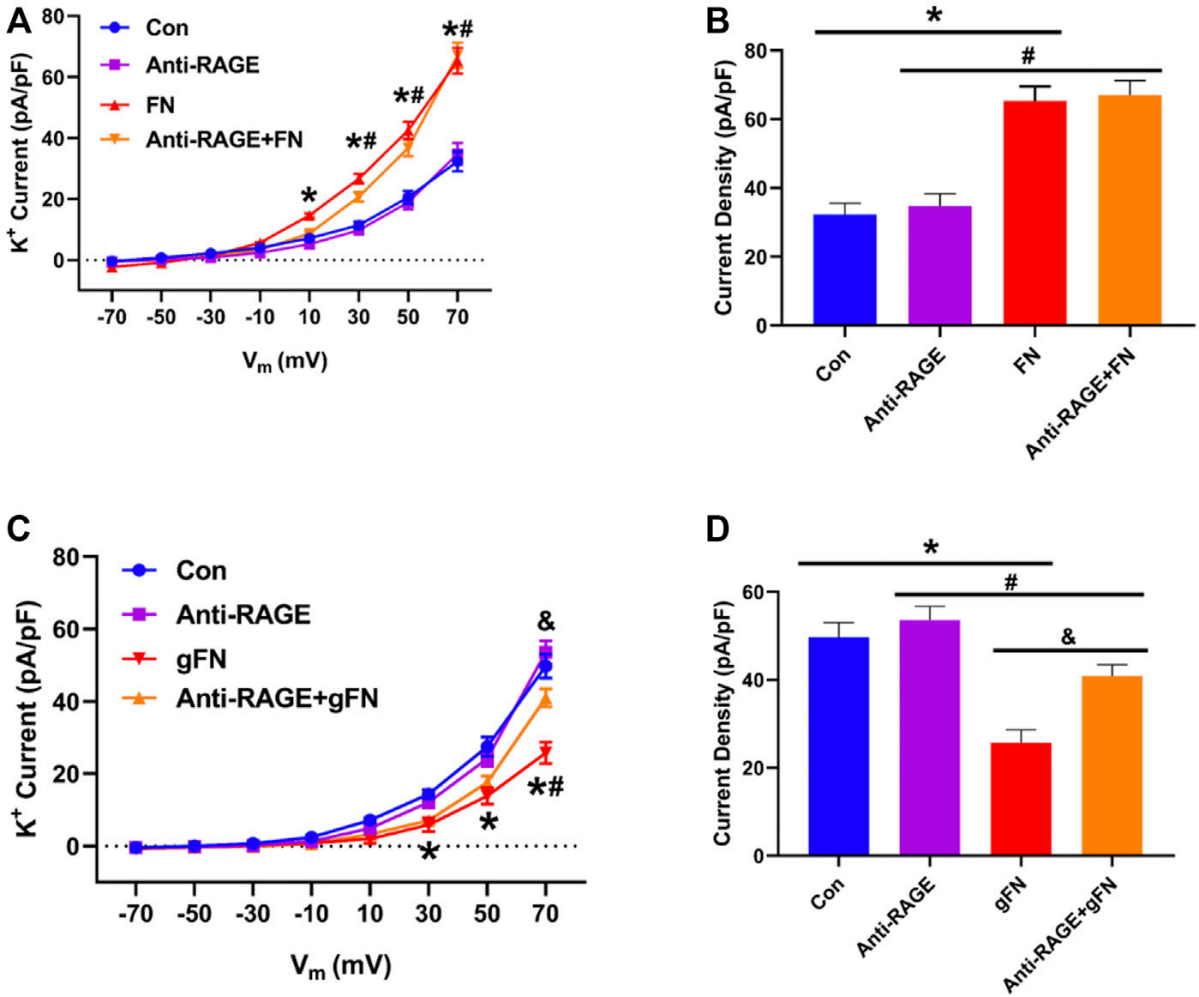
Effect of a function blocking anti-RAGE antibody on FN and gFN-mediated changes in whole cell K^+^ current. Panel **(A)** Group data (*n* = 10–16) showing the effect of anti-RAGE antibody on current density—voltage relationships under control conditions and in response to FN. Panel **(B)** Summary of data from Panel **(A)** recorded at a voltage step potential of +70 mV. Anti-RAGE antibody shows no effect on the stimulatory action of native FN on whole cell K^+^ current. Results are shown as mean ± SEM; *n* = 9–13 cells per group. *p < 0.05, Control vs. FN; #, Anti-RAGE vs. Anti-RAGE + FN. Panel **(C)** Group data (*n* = 12–15) showing the effect of anti-RAGE antibody on current density—voltage relationships under control conditions and in response to gFN. Panel **(D)** Summary of data from Panel **(C)** at a holding potential of +70 mV. Anti-RAGE antibody significantly attenuated the inhibitory effect of gFN on whole cell K^+^ current. *p < 0.05, Control vs. gFN; #*p* < 0.05, Anti-RAGE Vs. Anti-RAGE + gFN; & *p* < 0.05, gFN vs. Anti-RAGE + gFN. Results are shown as mean ± SEM.

Treatment of freshly isolated cerebral artery smooth muscle cells with a function blocking anti-RAGE antibody (100 μg/ml) partially prevented the inhibitory effects of gFN on K^+^ channel activity, returning the whole cell current towards baseline (Figure 7). The antibody, however, exerted no effect on native fibronectin’s ability to potentiate K^+^ channel current (Figure 7).

### 3.5 Glycated Fibronectin Induced ROS Generation

**FIGURE 8:**
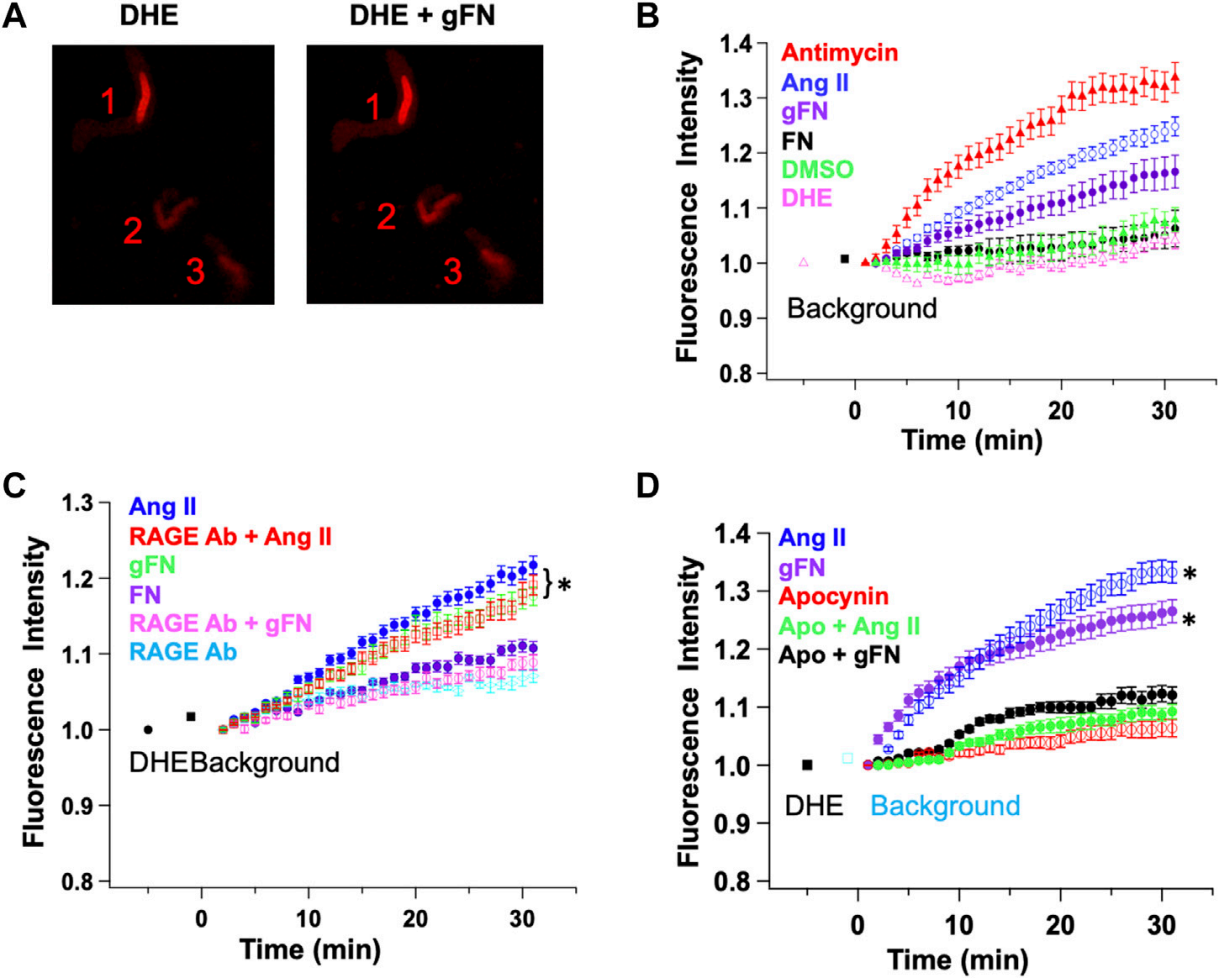
gFN causes production of reactive oxygen species (ROS) in cerebral VSMCs as measured by dihydroethidium (DHE) fluorescence. Panel **(A)** Representative confocal images before (DHE) and after (DHE + gFN) exposure of VSMCs to gFN. Panel **(B)** Time-dependent increase in ROS production is significantly greater in response to gFN (*n* = 6) compared with native FN (*n* = 3). Antimycin (10^−5^ M; *n* = 8) and angiotensin II (Ang II; 10-^7^M; *n* = 6) were used as positive controls for *de novo* ROS generation. DMSO treatment alone was used as a vehicle control. Panel **(C)** ROS production in response to gFN was attenuated by anti-RAGE antibody while that occurring in response to Ang II was not. RAGE antibody alone did not alter time-dependent increases in DHE fluorescence. N = 5–11 cells per condition. Panel **(D)** The NADPH oxidase inhibitor apocynin (5 × 10^−4^ M) reduced ROS production in response to both gFN and Ang II. N = 5–9 cells per condition. Results are shown as mean ± SEM. *p < 0.05 (vs. RAGE Ab or treatment in the presence of apocynin).

ROS species have been shown to be generated in response to RAGE ligation and to modulate the activity of ion channels (Mawatari et al., 2008; Win et al., 2012). ROS generation by freshly isolated cerebral artery SMCs in the presence of gFN was thus monitored using DHE fluorescence and confocal microscopy. As positive controls, ROS generation was also examined in response to antimycin (Heumuller et al., 2008; Gao et al., 2009) and angiotensin II (Ang II) (Touyz and Schiffrin, 2001). Example images of isolated VSMCs showing ROS-induced increases in DHE fluorescence are shown in Figure 8A.

In isolated cerebral VSMCs, both antimycin (10^−5^ M) and angiotensin (10^−7^ M) caused a time-dependent increase in DHE fluorescence (Figures 8B–D). This effect was significantly greater than control cells treated with DHE alone or DMSO (vehicle for Ang II and DHE). It should be noted that the control cells did show a minor increase in fluorescence, presumably representing a basal production of ROS in all cells. gFN caused a detectable increase in ROS accumulation, which was significantly greater than cells under control conditions (Figure 8).

To determine whether ROS generation occurred downstream of RAGE ligation, cells were treated with gFN in either the absence or presence of a function blocking RAGE antibody. As shown in Figure 8, the anti-RAGE antibody attenuated the gFN-induced increase in DHE fluorescence. In contrast, the anti-RAGE antibody had no apparent effect on the increase in fluorescence induced by Ang II (Figure 8 C) nor did it alter the baseline production of ROS as indicated by the time dependent increase in fluorescence in control cells (Figure 8).

Additional groups of cells were treated with the NADPH oxidase and ROS generation inhibitor, apocynin (5 × 10^−4^ M), as Ang II-induced increases in DHE fluorescence have been shown previously to be attenuated by this agent (Touyz and Schiffrin, 2001; Gao et al., 2009) Consistent with a possible role for NADPH oxidase and ROS, apocynin treatment decreased the time-dependent increase in fluorescence in cells stimulated with either Ang II or gFN **(** Figure 8D **)**.

## 4 Discussion

Physical interactions between ECM proteins and cells of the vascular wall underlie a bidirectional communication system that allows cells to respond to environmental stimuli (for example, changes in shear and stretch), transmit intracellular mechanical forces to the ECM and alter adhesive properties through inside-out signaling (Sun et al., 2008; Hong et al., 2012; Sun et al., 2012; Hong et al., 2014). Such events are believed to contribute to the functional behavior of the vascular wall, including acute vasomotor responsiveness that engages the activities of multiple vascular ion channels, and more chronic features of vascular remodeling and growth. In the present study, we hypothesized that modification of ECM proteins *via* non-enzymatic glycation may impact these functions and that ultimately, this process may provide a mechanism that could contribute to vascular dysfunction in disease states such as diabetes.

In the present study, *in vitro* protein non-enzymatic protein glycation was undertaken using methylglyoxal. Methylglyoxal is recognized as a physiologically relevant metabolite (Shah and Brownlee, 2016) and has been used as a glycating agent in a number of studies (for example, (Ahmed et al., 2003; Thornalley et al., 2003; Cantero et al., 2007). Mass spectrometry studies have demonstrated that protein glycation with methylglyoxal leads to the formation of AGEs including carboxymethyl and carboxyethyl lysine (Cantero et al., 2007; Pastino et al., 2017) which are also produced in the presence of agents such as glucose (Naser et al., 2013) and glucose-6-phosphate (Naser et al., 2013). In relation to methylglyoxal-induced glycation of fibronectin, using a mass spectrometry approach, Pastino et al. reported that methylglyoxal treatment led to the glycation of 28 sites within the FN fragment II_9-10_. Importantly, these sites included arginine residues within presumably functional integrin binding RGD sequences (Pastino et al., 2017). As a consequence, glycation may be expected to impact the functional properties of normal fibronectin binding. In addition, increased AGE formation would be expected to increase binding to RAGE, leading to enhanced signal transduction, including generation of ROS, through this pathway. Consistent with our previous study, using atomic force microscopy, we reported an apparent shift in fibronectin binding from vascular smooth muscle cells towards ligation of RAGE (Dhar et al., 2017). Also in agreement with this observation, the present study demonstrates increased gFN-induced ROS formation in VSMC that was attenuated by anti-RAGE antibody and impaired integrin-mediated modulation of K^+^ channels. We did not, however, extensively characterize fibronectin glycation sites at the molecular level.

As illustrated in Figures 1–4, extensively post-translationally modified proteins, gFN and gALB, attenuated whole cell K^+^ currents as measured in freshly isolated cerebral VSMCs by conventional patch clamp recording. It is well established that vascular K^+^ channels underlie vasodilator responses, limit vasoconstriction and, hence, contribute to the regulation of vascular tone (Nelson and Quayle, 1995; Ledoux et al., 2006; Hill et al., 2010; Tykocki et al., 2017). Importantly, glycation of fibronectin abolished the stimulatory effect on K^+^ current observed when smooth muscle cells are exposed to native fibronectin (Figures 1, 2, 4). FN stimulation of SMC K^+^ channels has previously been shown to be mediated through the ligation of integrins including those comprised of alpha 5 and beta 1 subunits (Gui et al., 2010; Chao et al., 2011; Wu et al., 2020). Albumin was used as a non-integrin dependent protein that would be expected, upon glycation, to initiate interactions with RAGE similar to other proteins, including gFN.

The effect of AGE proteins on macroscopic K^+^ currents in isolated VSMCs does not appear to be limited to a single class of channels. Thus, it appeared that both smooth muscle cell voltage-gated K^+^ channels (4-aminopryidine-sensitive) and large conductance, Ca^2+^-activated, K^+^ channels are affected. It should be noted that other classes of K^+^ channels (including K_ATP_, K_V7_, two pore K^+^ channels), which are present in small artery smooth muscle cells, were not specifically examined in this study. An interesting question is whether the glycated forms of fibronectin and albumin exert their effects on K_V_ and BK_Ca_ channels through similar or differing mechanisms. In support of a similar cellular mechanism, both classes of K^+^ channel have been shown to be modulated by ROS (Li et al., 2004; Tang et al., 2004; Mittal et al., 2012; Hua et al., 2022). Increased ROS production in response to RAGE activation is supported by the results of the present study in VSMCs as well as reports from other cellular systems (Wautier et al., 2001; Fukami et al., 2004; Manigrasso et al., 2014; Piras et al., 2016). Further, RAGE activation leads to an NFκB-mediated increase in RAGE expression, resulting in further ROS production (Ramasamy et al., 2005; Bongarzone et al., 2017). Although the exact source or species of ROS leading to attenuation of K^+^ channel currents by AGE proteins was not investigated in the present study, we speculate that NADPH oxidase and hydrogen peroxide were involved. This is based on the ability of apocynin to inhibit AGE protein-induced ROS production (Figure 8) and that the fluorescent indicator DHE readily detects cellular superoxide anions (Nauseef, 2014). However, as apocynin has been reported to exert effects independent of NADPH inhibition, including acting as a ROS scavenger, further studies are required (Heumuller et al., 2008; Rosa et al., 2016).

While native fibronectin was shown to increase macroscopic K^+^ channel currents, gFN decreased these activities. BK_Ca_ current activation was evident through the ability of fibronectin to increase iberiotoxin-sensitive STOCs (spontaneous transient outward currents reflecting the activity of a cluster of BK_Ca_ channels) (Figure 4). In contrast, gFN did not stimulate macroscopic K^+^ currents or increase STOC activity (Figures 1, 2, 4). These effects appeared to be relatively specific for gFN and not a general response to glycated proteins as gALB exerted only a comparatively modest inhibitory effect on the frequency of STOCs while native albumin had no apparent effect on the appearance of STOCs. We speculate that the inhibitory effect of gFN on STOCs is predominantly mediated through a block of integrin-mediated modulation of the BK_Ca_ channel, as suggested by the data shown in Figures 6, 7. In an earlier study, native FN was reported to enhance BK_Ca_ channel activity through a Src kinase/tyrosine phosphorylation– dependent mechanism (Yang et al., 2010). Importantly, BK_Ca_ channels have been shown to be the ion channels underlying STOC activity in vascular smooth muscle cells and to provide a mechanism for regulation vascular tone and responses to agonists (Nelson and Quayle, 1995; Ledoux et al., 2006; Yang et al., 2009; Hill et al., 2010; Tykocki et al., 2017).

A caveat on the approach presented here relates to the study of glycated ECM protein cell interactions with VSM not chronically exposed to hyperglycemia as would be expected in the situation of poorly controlled diabetes. While we chose this approach to isolate the effects of glycated proteins *per se*, under conditions of chronic exposure of the VSMCs to hyperglycemia or glycated proteins, additional differences may be expected at the level of RAGE expression and conceivably integrin and/or ion channel protein expression. As mentioned above this possibility is supported by previous studies showing that AGE-modified proteins upregulate RAGE expression through NFκB-dependent mechanisms (Ramasamy et al., 2005; Bongarzone et al., 2017). Despite this, it is important to note that the native vascular cells used in these studies express both RAGE (see Supplementary Material) and fibronectin-binding integrins (Sun et al., 2005; Sun et al., 2008). As hyperglycemia *per se* has also been reported to inhibit BK_Ca_ activity in rat coronary arteries through ROS-dependent mechanisms (Zhang et al., 2020) it is likely that under *in vivo* conditions of chronic hyperglycemia, disturbances in vascular smooth muscle K^+^ channel function/regulation may be exacerbated. Further, in regard to intact arteries, inhibition of endothelial cell nitric oxide production by glycated proteins/ROS (Naser et al., 2013) would be expected to contribute to impaired vasodilation.

A further limitation is that the studies described in this report relied largely on a pharmacological approach using single concentrations of function blocking antibodies in rat VSMCs. Future studies should consider the use of genetically modified mice, for example, cell-specific RAGE and integrin subunit knockout models. Our experiments related to Kv currents employed a widely-used, broad spectrum, inhibitor (4-AP). More in-depth studies will be required to further dissect the specific components of the 4-AP inhibitable currents as will be studies to determine the impact of glycated proteins on ion channel kinetics. Finally, the current studies were performed only on cells isolated from male rats. Possible sexually dimorphic findings cannot be excluded and should be determined in future studies.

In conclusion, the present studies identify ion channel-related mechanisms by which protein glycation may modify VSMC function (see Supplementary Figure S5 for a schematic representation). Notably, non-enzymatic glycation of the ECM protein, fibronectin, changed its modulatory actions on VSMC K^+^ channel activity from that of a stimulant to an inhibitor. Such a change in action would be predicted to impair vasodilation. Dysregulation of K^+^ channels was shown to result from disruption of physiological mechanisms, in particular the regulation of BK_Ca_ channel activity by integrin-ECM interactions, along with the pathological generation of ROS by gFN.

## Data Availability

The raw data supporting the conclusion of this article will be made available by the authors, without undue reservation.
