# Supplementary figures and images for: Modification of Fibronectin by Non-Enzymatic Glycation Impairs K+ Channel Function in Rat Cerebral Artery Smooth Muscle Cells
Source: Front Physiol. 2022 Jun 27;13:871968. doi: 10.3389/fphys.2022.871968 (PMC9272009; doi:10.3389/fphys.2022.871968)

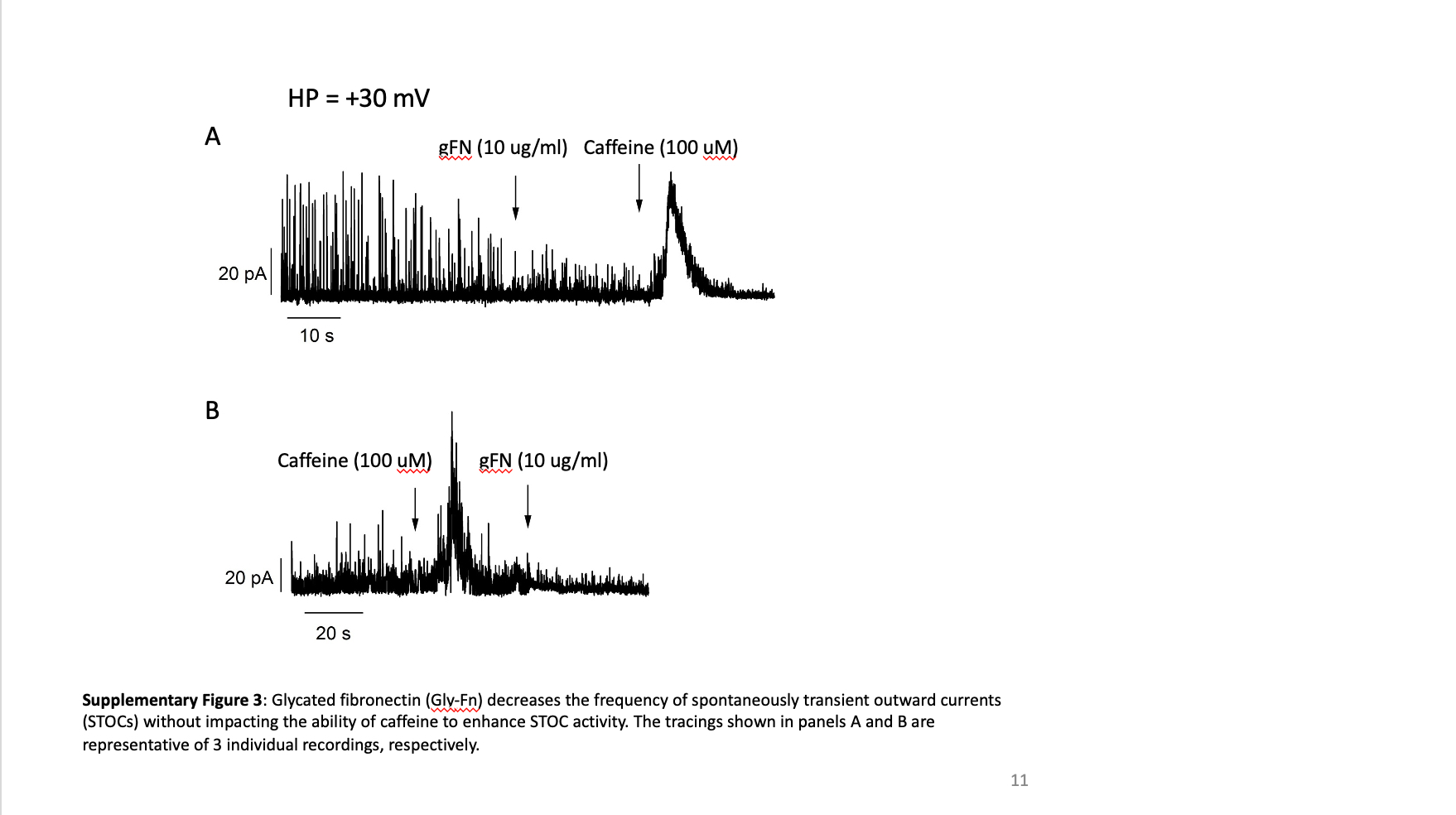

Supplement: Supplementary file 1 [file Image3.JPEG]

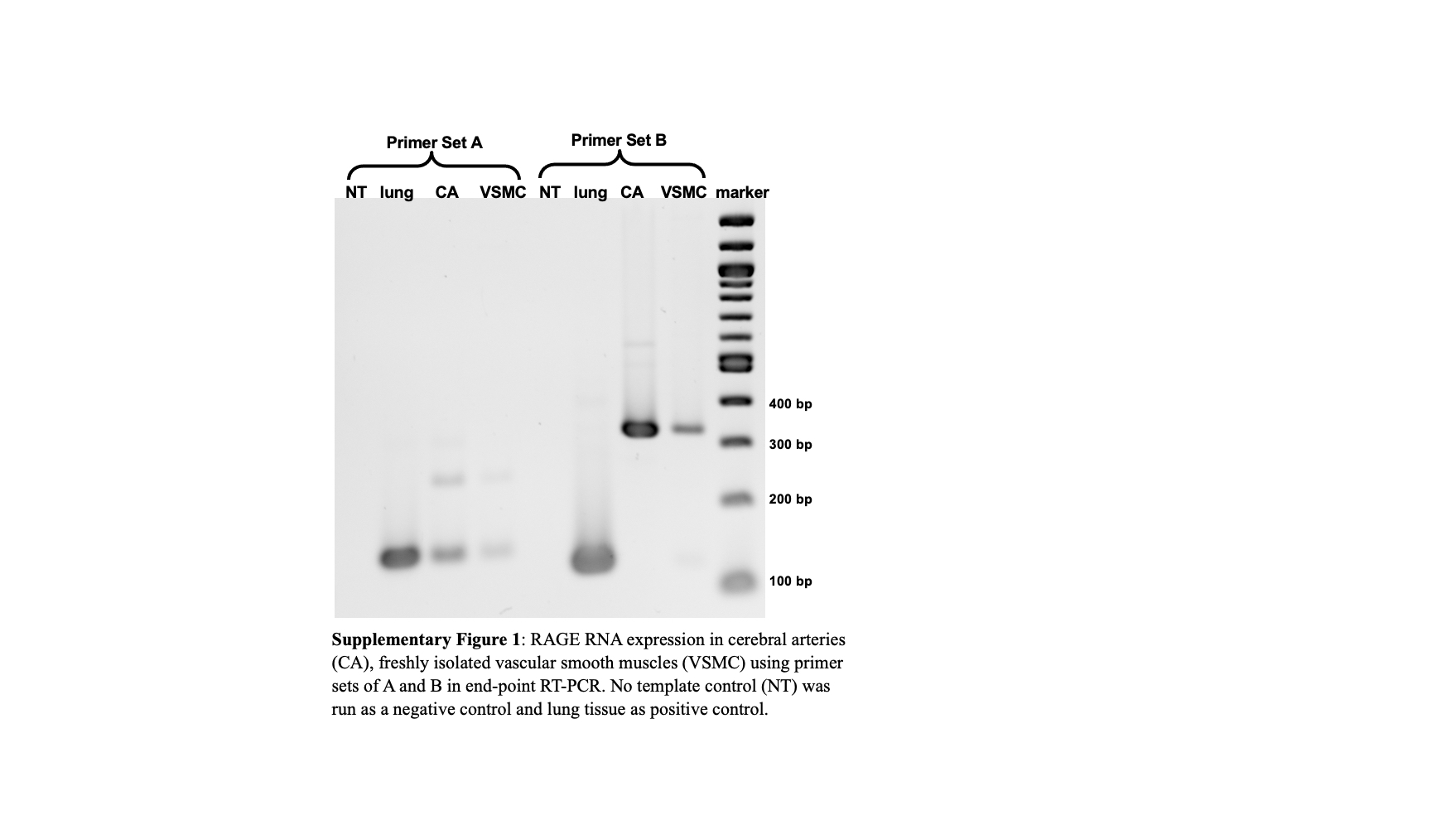

Supplement: Supplementary file 2 [file Image1.JPEG]

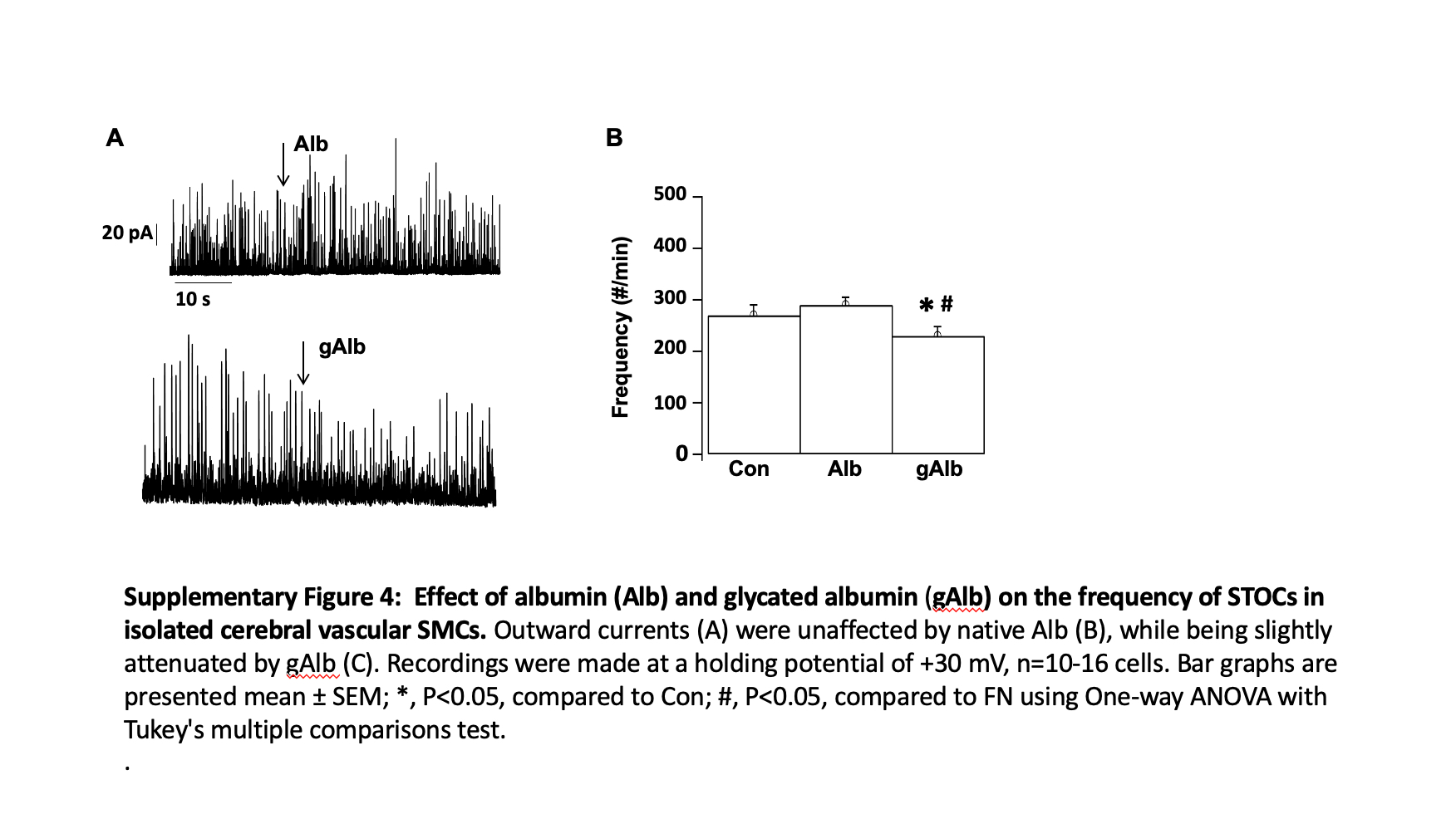

Supplement: Supplementary file 3 [file Image4.JPEG]

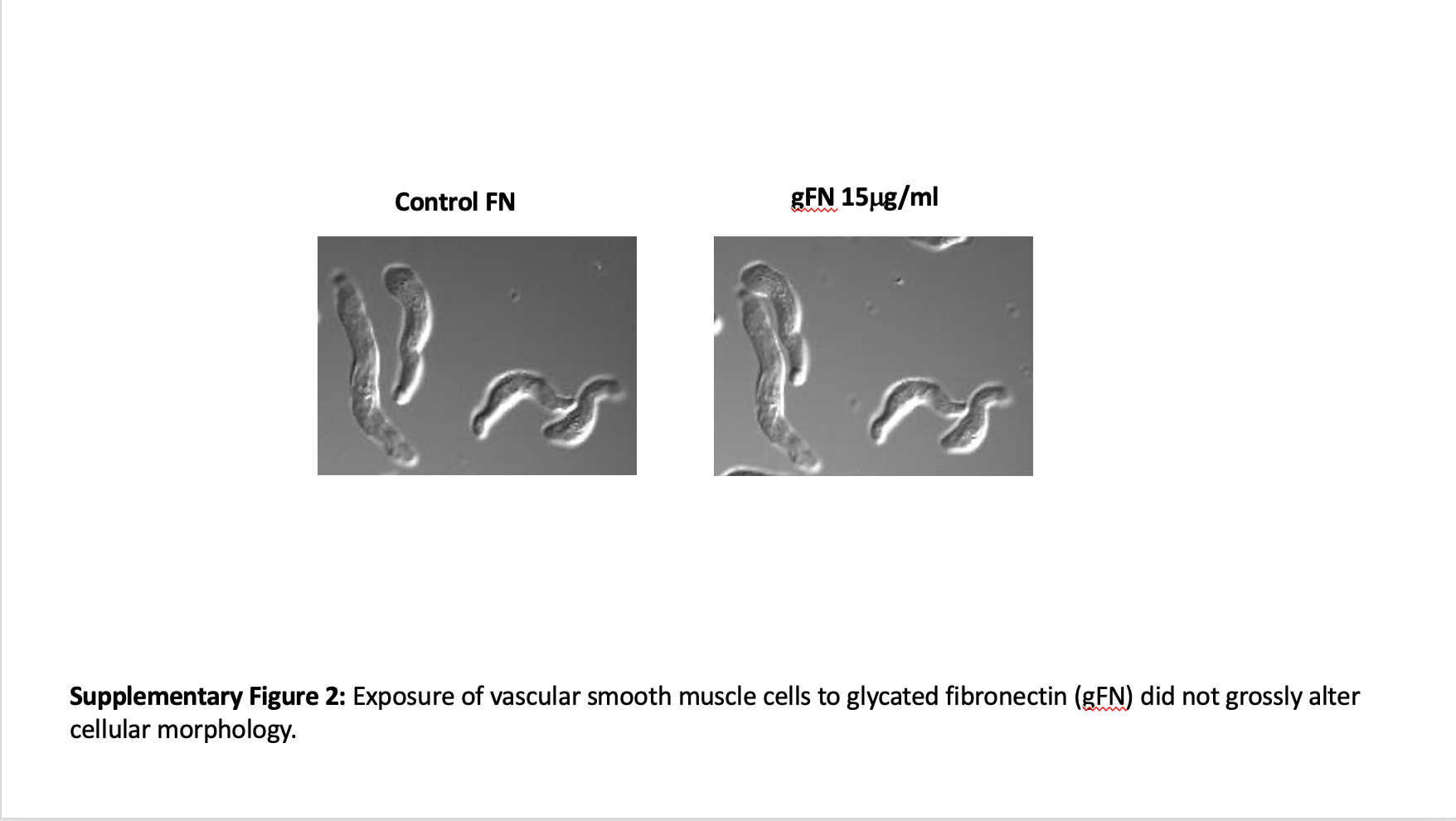

Supplement: Supplementary file 4 [file Image2.JPEG]

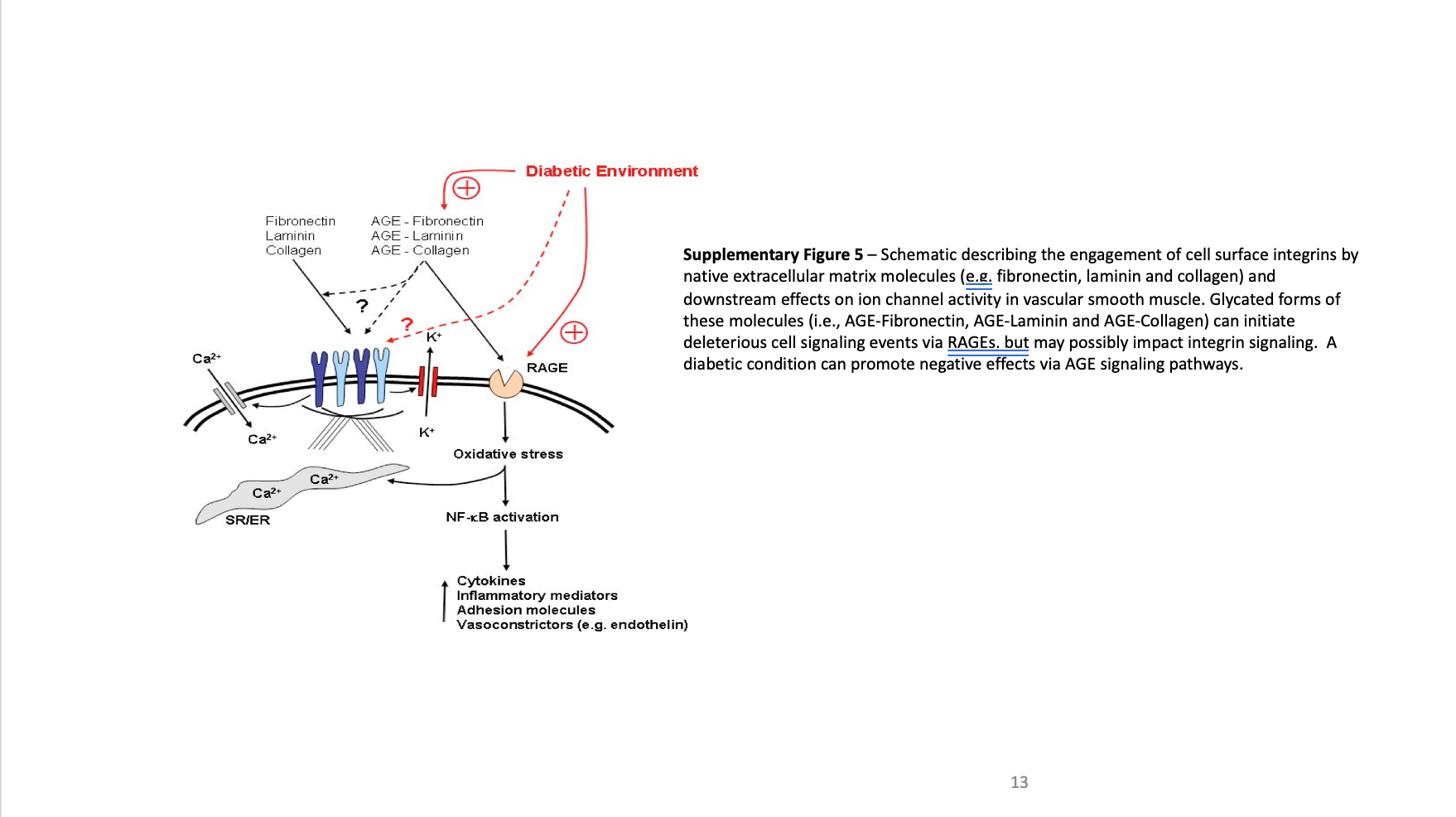

Supplement: Supplementary file 5 [file Image5.JPEG]
